# Mechanistic and structural elucidation of π–π end-stacking interactions of a novel SO_2_NH-anthraquinone derivative with telomeric G-quadruplex d-[TTAGGGT]_4_: integrated NMR, spectroscopic, electrochemical, and computational evidence

**DOI:** 10.1039/d6ra02891h

**Published:** 2026-07-11

**Authors:** Ravinder Sharma, Ritu Kundu, Arti Sharma, Manu Vatsal, Pamita Awasthi

**Affiliations:** a Department of Chemistry, National Institute of Technology Hamirpur HP 177005 India sharmaravinder444@gmail.com pamita@nith.ac.in pamitawasthi@gmail.com

## Abstract

Targeting telomeric G-quadruplex (G4) DNA has emerged as a promising anticancer strategy by inhibiting telomerase activity and disrupting telomere maintenance. In the present study, we report a comprehensive structural, spectroscopic, electrochemical, and computational investigation of a novel anthracene-9,10-dione derivative, *N*-(9,10-dioxo-9,10-dihydroanthracen-1-yl)-3-methyl-2-(phenylsulfonamido)butanamide (1-VAQ), and its interaction with the parallel G-quadruplex d-[TTAGGGT]_4_. To evaluate binding selectivity, comparative studies were also performed using duplex DNA as a control. ^1^H NMR spectroscopy revealed localized chemical shift perturbations and exchange-dependent line broadening upon complex formation, providing direct evidence of ligand–DNA interaction. Circular dichroism studies demonstrated preservation of the characteristic G-quadruplex topology accompanied by concentration-dependent enhancement of ellipticity, whereas significantly smaller perturbations were observed for duplex DNA. UV-visible absorption and fluorescence titrations revealed markedly stronger binding of 1-VAQ toward d-[TTAGGGT]_4_ than duplex DNA, with G-quadruplex binding constants in the order of 10^6^ M^−1^ compared with 10^4^ M^−1^ for duplex DNA. Electrochemical studies further supported preferential G-quadruplex recognition, yielding binding constants of 2.87 × 10^5^ M^−1^ and 1.50 × 10^4^ M^−1^ for G-quadruplex and duplex DNA, respectively. Thermodynamic analysis afforded highly favorable Gibbs free energy values for G-quadruplex binding (Δ*G* = −31 to −36 kJ mol^−1^), indicating a spontaneous and energetically preferred interaction relative to duplex DNA. Dynamic light scattering (DLS) confirmed the formation of stable ligand–DNA complexes in solution. Molecular docking and DFT calculations corroborated the experimental findings, revealing energetically favorable association of the anthraquinone chromophore with the G-quadruplex surface, supported by groove-directed hydrogen bonding and electrostatic interactions. Collectively, the spectroscopic, electrochemical, thermodynamic, and computational results demonstrate that 1-VAQ exhibits pronounced selectivity toward d-[TTAGGGT]_4_ over duplex DNA, establishing this anthracene-based scaffold as a promising platform for the development of G-quadruplex-targeted anticancer agents.

## Abstract 2

Interaction of G-quadruplex (G4) DNA with anthracene-9,10-dione derivative, *N*-(9,10-dioxo-9,10-dihydroanthracen-1-yl)-3-methyl-2-(phenylsulfonamido)butanamide (1-VAQ).

## Graphical abstract

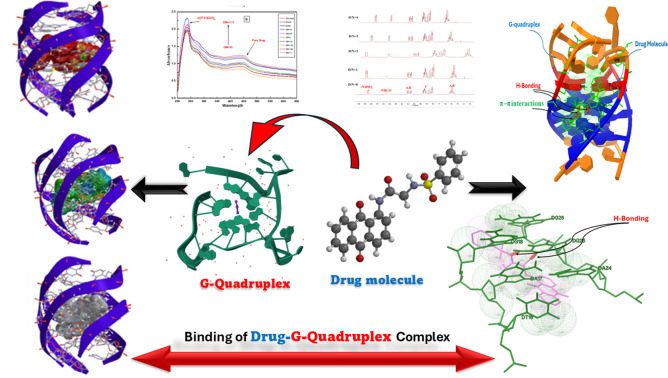

## 1. Introduction

G-Quadruplex (G4) DNA is a unique non-canonical secondary structure formed by guanine-rich sequences, where four guanine bases connect *via* Hoogsteen hydrogen bonds to create planar guanine tetrads. These tetrads stack on top of each other. They are stabilized by monovalent cations, such as K^+^ or Na^+^, resulting in highly stable four-stranded structures that differ significantly from the traditional B-DNA double helix.^1–5^ The structural flexibility of G-quadruplexes, which can appear as parallel, antiparallel, or hybrid forms depending on the sequence and ion conditions, underpins their various biological functions in maintaining genome integrity and regulating gene expression.^6–9^

At telomeres, the formation of G-quadruplexes prevents uncontrolled chromosome extension by restricting telomerase access.^10–13^ Overexpression of telomerase, a common feature in many cancers, allows for unlimited cell replication. Therefore, stabilizing telomeric G-quadruplexes has become an attractive anticancer approach: the folded structures make telomeric DNA inaccessible to telomerase, leading to gradual telomere shortening and eventual cell death.^14–18^ Additionally, G-quadruplex-forming sequences are abundant in the promoter regions of oncogenes such as c-MYC (MYC proto-oncogene), KRAS (Kirsten rat sarcoma viral oncogene homolog), and VEGF (vascular endothelial growth factor), where their stabilization can suppress transcription and limit tumor growth.^19–26^ As a result, ligands that recognize and stabilize G-quadruplexes present a promising strategy to target both telomerase activity and oncogenic transcription pathways.

Among known G-quadruplex binders, anthraquinone derivatives have attracted significant interest due to their planar aromatic structures and established anticancer properties.^27–36^ Their π-conjugated cores facilitate stacking interactions with terminal guanine quartets, while side chains designed for optimal binding enhance groove or loop interactions through electrostatic and hydrogen bonds. These properties enable anthraquinone analogues to act as dual-function agents, disrupting DNA topology and stabilizing G-quadruplexes, thereby providing mechanistic versatility for the development of anticancer agents.^37–43^

Building on this rationale, we synthesized a new anthraquinone derivative, 1-VAQ, incorporating a sulfonamide moiety to improve solubility and facilitate DNA recognition. Preliminary cytotoxicity studies in the Hep2C (HeLa) cell line revealed significant inhibition of cell viability, with activity comparable to the clinically established agent mitoxantrone.^42–44^ Furthermore, 1-VAQ demonstrated cell cycle perturbation, particularly affecting progression from the G1 to G2 phases. The compound appears to interfere with S-phase progression, thereby disrupting DNA replication, a process essential for cellular proliferation.^45–50^

To explore the molecular basis of its anticancer activity and evaluate its DNA-binding selectivity, we investigated the interaction of 1-VAQ with the parallel telomeric G-quadruplex d-[TTAGGGT]_4_ and compared its binding behavior with duplex DNA using an integrated spectroscopic, electrochemical, and computational approach. ^1^H NMR spectroscopy was employed to obtain molecular-level information regarding ligand–DNA interactions through chemical shift perturbation analysis, while circular dichroism (CD) spectroscopy was used to examine ligand-induced structural changes and topology preservation. UV-Vis absorption and fluorescence spectroscopy were utilized to determine binding affinity, stoichiometry, and the nature of the interaction in both DNA systems. Cyclic voltammetry provided complementary electrochemical evidence of complex formation and enabled quantitative evaluation of binding strength, whereas dynamic light scattering (DLS) monitored changes in hydrodynamic size associated with ligand–DNA complexation. In addition, molecular docking and density functional theory (DFT) calculations were performed to elucidate the preferred binding geometry, interaction energies, and electronic factors governing complex stability. By directly comparing the interactions of 1-VAQ with G-quadruplex and duplex DNA, this study provides a comprehensive assessment of its binding selectivity and molecular recognition properties. The combined experimental and computational results offer important insights into the potential of anthracene-9,10-dione-based scaffolds as selective G-quadruplex-targeting agents for anticancer applications.

## 2. Materials and methods

### 2.1 Materials and reagents

The oligonucleotide sequence d-(TTAGGGT) was purchased in desalted form from Merck (Germany) and used for the preparation of the tetramolecular G-quadruplex d-(TTAGGGT)_4_. Duplex DNA (ct-DNA) was used as a non-G-quadruplex control to evaluate the ligand's binding selectivity across different DNA topologies. Potassium chloride (KCl), dipotassium hydrogen phosphate (K_2_HPO_4_), and ethylenediaminetetraacetic acid (EDTA) were used for the preparation of a 10 mM potassium phosphate buffer containing 100 mM KCl. All chemicals and reagents were of analytical grade and were used as received without further purification. Ultrapure water was used throughout all experiments. Stock solutions of DNA were prepared in the buffer system and stored at 4 °C prior to use. The concentration of DNA was determined spectrophotometrically using the appropriate molar extinction coefficients. All experiments were performed under identical buffer conditions to enable direct comparison between G-quadruplex and duplex DNA binding.

### 2.2 G-Quadruplex[d-(TTAGGGT)]_4_ preparation

The oligonucleotide sequence d-(TTAGGGT) was prepared in a 10 mM KBPES buffer (pH 7.0) containing 10 mM K_2_HPO_4_, 1 mM EDTA, and 100 mM KCl; including KCl in the buffer was essential, as potassium ions are well-known for their role in stabilizing G-quadruplex structures by coordinating with guanine tetrads, thereby increasing the structural stability of the folded oligonucleotide. A controlled thermal annealing process ensured the proper folding of d-(TTAGGGT) into a stable G-quadruplex conformation. The oligonucleotide solution was initially heated to 90 °C for 5 minutes, a step designed to break any pre-existing secondary structures or intermolecular interactions that may have formed during preparation. The sample was then allowed to cool slowly to room temperature overnight. This gradual cooling was crucial, as it enabled proper alignment and stacking of guanine tetrads, thereby promoting the formation of a well-defined, thermodynamically stable G-quadruplex structure. After annealing, the sample was stored at 4 °C to preserve the integrity of the quadruplex conformation until further analysis. This storage condition was chosen to minimize structural disturbances and ensure the reproducibility of biophysical and spectroscopic data measurements.^51–53^ The concentration of the G-quadruplex-forming oligonucleotide [d-(TTAGGGT)]_4_ was precisely measured using ultraviolet-visible (UV-Vis) spectroscopy, a widely recognized and dependable method for quantifying nucleic acids. UV-Vis spectroscopy provides a non-destructive and highly consistent method for determining oligonucleotide concentration by measuring absorbance at a specific wavelength, typically 260 nm, where nucleic acid bases strongly absorb due to their conjugated π-electron systems. To ensure accurate concentration measurement, the molar absorptivity (*ε*) at 260 nm was estimated by summing the individual extinction coefficients of its nucleotides: thymidine (dT), deoxyguanosine (dG), and deoxyadenosine (dA). These coefficients were obtained from established tables and adjusted for the oligonucleotide sequence composition. Using these values, the concentration of the folded G-quadruplex was calculated with the Beer–Lambert law: *A* = *εcl*, where *A* is the measured absorbance, *ε* is the molar absorptivity (L mol^−1^ cm^−1^), *c* is the oligonucleotide concentration (mol L^−1^), and *l* is the cuvette's optical path length, which was set at 1 cm for all measurements.

### 2.3 Nuclear magnetic resonance (NMR) binding analysis

^1^H NMR experiments were performed to investigate the interactions of 1-VAQ with both the tetramolecular G-quadruplex d-(TTAGGGT)_4_ and duplex DNA in order to evaluate ligand binding and selectivity toward different DNA topologies. Spectra were recorded on a JEOL JNM-ECS400 FT NMR spectrometer operating at 400 MHz. Samples were prepared in NMR-compatible buffer conditions, and spectra were acquired at ambient temperature. For the G-quadruplex studies, titrations were performed by the gradual addition of 1-VAQ to pre-folded d-(TTAGGGT)_4_, and changes in the imino and aromatic proton regions were monitored. Chemical shift perturbations, signal broadening, and intensity changes were analyzed to identify ligand-induced alterations in the local magnetic environment of the quadruplex structure. Similar experiments were performed using duplex DNA under identical conditions to provide a direct comparison of binding behavior. The observed spectral changes were used to assess the strength and nature of ligand–DNA interactions and to distinguish the preferential association of 1-VAQ with the G-quadruplex relative to duplex DNA. Comparative analysis of the NMR spectra provided molecular-level information regarding ligand recognition, binding-induced perturbations, and DNA topology-dependent interactions.

### 2.4 Circular dichroism (CD) analysis

Circular dichroism (CD) spectroscopy was employed to investigate the structural characteristics of the G-quadruplex d-(TTAGGGT)_4_ and duplex DNA and to evaluate the influence of 1-VAQ binding on their respective DNA topologies. CD spectra were recorded at room temperature using a JASCO J-1500 CD spectropolarimeter (serial no. A006361638). All measurements were performed in 10 mM phosphate buffer (pH 7.4) containing 100 mM KCl under conditions identical to those used in the UV-visible absorption and fluorescence studies. Spectra were acquired using a 1 mm path-length quartz cuvette over the wavelength range of 200–400 nm. For the titration experiments, a fixed concentration of DNA was maintained in the cuvette and increasing aliquots of 1-VAQ were added stepwise. Aliquots of 1-VAQ solution (2 µL per addition) were added stepwise to generate a series of *D*/*N* ratios, allowing the effect of increasing DNA concentration on the ligand–DNA system's CD response to be monitored. After each addition, the solution was gently mixed and allowed to equilibrate before spectral acquisition. Each spectrum represents the average of three consecutive scans to improve the signal-to-noise ratio. Baseline correction was performed using the corresponding buffer spectrum. Comparative analysis of the CD spectra obtained for d-(TTAGGGT)_4_ and duplex DNA was used to evaluate ligand-induced changes in ellipticity, preservation of topology, and the relative preference of 1-VAQ for different DNA architectures.

Thermal melting experiments were performed using a JASCO J-1500 circular dichroism spectropolarimeter equipped with a Peltier temperature controller. Ligand-bound samples were prepared by incubating the folded G-quadruplex with appropriate concentrations of 1-VAQ for 12 h at room temperature. Thermal denaturation profiles were recorded by monitoring the CD signal at 270 nm, corresponding to the characteristic positive band of the G-quadruplex structure. The temperature was increased from 20 to 90 °C at a heating rate of 1 °C min^−1^. The melting temperature (*T*_m_) was determined from the midpoint of the unfolding transition. The stabilization effect of 1-VAQ was evaluated from the change in melting temperature (Δ*T*_m_) according to:

1 Δ*T*_m_ = *T*_m,complex_ − *T*_m,free_

where *T*_m,complex_ and *T*_m,free_ represent the melting temperatures of the ligand-bound and free G-quadruplex, respectively.

### 2.5 UV-vis spectroscopic analysis

UV-visible absorption measurements were performed using a Shimadzu T80+ UV-Vis spectrophotometer (Japan) equipped with a Peltier-controlled thermostatic cell holder. All spectra were recorded in a 1 cm path-length quartz cuvette over the wavelength range of 200–800 nm at 25 °C. Baseline correction was carried out using the corresponding buffer solution under identical experimental conditions. To investigate the interaction of 1-VAQ with different DNA topologies, absorption titration experiments were performed using both the tetramolecular G-quadruplex d-(TTAGGGT)_4_ and duplex DNA. A fixed concentration of 1-VAQ (20 µM) was titrated with increasing concentrations of DNA while maintaining identical buffer conditions for both systems. The drug-to-DNA molar ratio (*D*/*N*), (for all titration experiments, the molar drug-to-DNA ratio (*D*/*N*), where *D* represents the concentration of 1-VAQ and *N* represents the concentration of DNA), was used to describe the relative proportions of ligand and nucleic acid. Was varied from 10 to 1.1 through successive additions of DNA solution. After each addition, the mixture was gently mixed and allowed to equilibrate for 5 min prior to spectral acquisition to ensure complete ligand–DNA interaction. Changes in absorbance intensity and spectral profile were monitored as a function of DNA concentration. The resulting titration data were analyzed to evaluate the binding affinity, binding stoichiometry, and selectivity of 1-VAQ toward G-quadruplex and duplex DNA. Binding constants were determined from the corresponding absorption titration plots using established spectroscopic binding models.

The equilibrium binding constant (*K*_b_) was determined quantitatively from absorbance titration data using a half-reciprocal plot, applying the standard binding equation.2
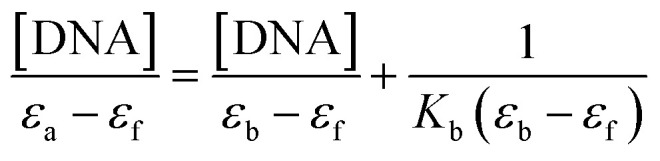
In this study, the concentration of the G-quadruplex [d-(TTAGGGT)]_4_ is represented as [DNA] = *N*. The apparent molar absorption coefficient (*ε*_a_) is defined as the ratio of the observed absorbance of the drug–DNA complex (*A*_obs_) to the fixed concentration of 1-VAQ ([*L*]), expressed as *ε*_a_ = (*A*_obs_/[*L*]). Furthermore, *ε*_f_ and *ε*_b_ correspond to the molar absorption coefficients of the drug in its free and DNA-bound states, respectively. The change in Gibbs free energy (Δ*G*) for the interaction between the drug and DNA was derived from the binding constant by applying the standard thermodynamic expression:

3 Δ*G* = −*RT* ln *K*_b_

where *R* is the universal gas constant (8.314 J mol^−1^ K^−1^) and *T* is the absolute temperature in Kelvin.

### 2.6 Fluorescence measurements

Fluorescence emission spectra were recorded using a Shimadzu RF-5301 PC spectrofluorometer (Japan) equipped with a xenon lamp excitation source. All measurements were performed at 25 °C under identical experimental conditions to enable direct comparison between G-quadruplex and duplex DNA binding. The fluorescence studies were carried out using the same samples employed in the UV-visible absorption experiments. The excitation wavelength (*λ*_ex_) was fixed at 290 nm, and emission spectra were collected over 300–700 nm using a 1 cm path-length quartz cuvette. To investigate the interaction of 1-VAQ with different DNA topologies, fluorescence titration experiments were performed using both the tetramolecular G-quadruplex d-(TTAGGGT)_4_ and duplex DNA. A fixed concentration of 1-VAQ was titrated with increasing concentrations of DNA, generating a series of *D*/*N* ratios identical to those used in the UV-visible absorption and CD studies. After each addition, the samples were gently mixed and allowed to equilibrate before fluorescence measurements. Changes in fluorescence intensity and emission profile were monitored as a function of DNA concentration to evaluate ligand–DNA interactions.

To quantitatively evaluate the binding interaction between 1-VAQ and the G-quadruplex [d-(TTAGGGT)]_4_, fluorescence quenching data were analyzed using the Stern–Volmer equation. The Stern–Volmer quenching constant (*K*_SV_) was calculated from the variation in fluorescence intensity with increasing DNA concentration. This approach provided critical insights into the quenching mechanism, enabling determination of the nature and strength of the interaction between the drug and the G-quadruplex. By integrating fluorescence and absorbance spectroscopic data, a more comprehensive understanding of the drug–DNA interaction was achieved, reinforcing the reliability of the binding analysis.4
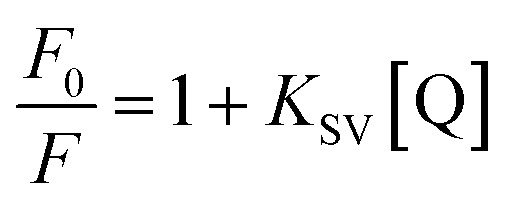
In this analysis, *F*_0_ and *F* represent the fluorescence intensities of 1-VAQ in its unbound (free) state and DNA-bound state, respectively. The quencher concentration ([Q]) corresponds to the concentration of the G-quadruplex DNA, specifically [d-(TTAGGGT)]_4_, which interacts with 1-VAQ, leading to fluorescence quenching. This quenching effect is a key indicator of the binding interaction between the drug and the G-quadruplex structure.

### 2.7 Electrochemical characterization *via* cyclic voltammetry

Cyclic voltammetry (CV) experiments were performed using a Metrohm Autolab PGSTAT302N electrochemical workstation (Herisau, Switzerland) equipped with a conventional three-electrode system consisting of a glassy carbon working electrode (GCE), an Ag/AgCl reference electrode, and a platinum wire counter electrode. Prior to each measurement, the glassy carbon electrode was polished with alumina slurry, thoroughly rinsed with deionized water, and dried to ensure a clean and reproducible electrode surface. Electrochemical measurements were carried out in solutions containing 1-VAQ, TEMPO as the redox mediator, and potassium nitrate (KNO_3_) as the supporting electrolyte. All electrochemical experiments were performed under identical buffer and instrumental conditions to ensure reliable comparison of the binding behavior of 1-VAQ toward G-quadruplex and duplex DNA. The electrochemical response of free 1-VAQ was initially recorded, followed by titration with increasing concentrations of DNA.

To evaluate the selectivity of 1-VAQ toward different DNA topologies, separate titration experiments were performed using the tetramolecular G-quadruplex d-(TTAGGGT)_4_ and duplex DNA. Increasing aliquots of DNA stock solution were added stepwise to the electrochemical cell to generate a series of *D*/*N* ratios identical to those employed in the UV-visible absorption, fluorescence, and CD studies. After each addition, the solution was gently mixed and allowed to equilibrate prior to electrochemical measurement. Changes in anodic and cathodic peak currents, peak potentials, and overall voltammetric response were monitored as a function of DNA concentration.^54,55^

The resulting electrochemical data were analyzed to evaluate ligand–DNA interactions, determine binding constants, and compare the affinity of 1-VAQ toward G-quadruplex and duplex DNA. Comparative analysis of the voltammetric responses provided quantitative information regarding the strength, selectivity, and thermodynamic favorability of ligand binding to the two DNA architectures. The direct comparison between d-(TTAGGGT)_4_ and duplex DNA enabled assessment of DNA topology-dependent recognition and provided electrochemical evidence for the preferential binding of 1-VAQ toward the G-quadruplex structure. The binding constant (*K*_b_) for the drug–DNA interaction was derived from the cyclic voltammetry data using the Benesi–Hildebrand equation, a well-established model for quantifying molecular interactions in electrochemical studies.^56,57^5
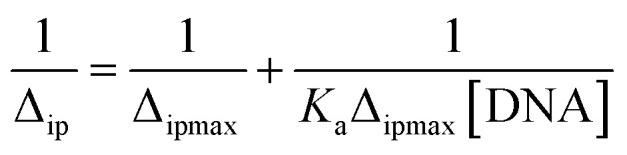
In this equation, Δ_ip_ = change in peak current (*i*_0_ − *i; i*_0_ and *i* are the peak current of the drug in the absence and presence of DNA [d-(TTGGGGT)]_4_ concentration. Δ_ipmax_ = maximum change in peak current, *K*_a_ = association (binding) constant.

### 2.8 Dynamic light scattering (DLS)

Dynamic Light Scattering (DLS), performed with a Malvern Zetasizer Version 6.2 (Serial no. MAL1049709), is a powerful biophysical technique for exploring the intricacies of drug–DNA interactions. It provides real-time, label-free insights into key parameters, including binding mechanisms, structural stability, electrostatic interactions, and aggregation behavior, by accurately measuring changes in hydrodynamic size and surface charge upon drug binding. This analytical capability is crucial for the rational design of DNA-targeting drugs, ensuring optimal stability, selectivity, and efficiency. Importantly, its ability to evaluate colloidal stability and aggregation tendencies is a game-changer for developing nanoparticle-based drug delivery systems, enabling researchers to fine-tune formulations for improved pharmacokinetics and therapeutic outcomes. Ultimately, the zeta potential profile serves as a blueprint for designing effective and stable therapeutic molecules. Beyond fundamental binding aspects, dynamic light scattering (DLS) is crucial for evaluating aggregation behavior and colloidal stability, especially in nanoparticle-based drug delivery systems. Managing surface charge and ensuring the dispersion of these nanoparticles are essential for their cellular uptake, circulation time, and overall bioavailability.

### 2.9 Density functional theory (DFT) analysis

To gain a detailed understanding of the structural and electronic properties of the anticancer drug 1-VAQ and its interaction with G-quadruplex DNA [d-(TTAGGGT)]_4_, density functional theory (DFT) calculations were performed using Spartan 20 version 1.1.4 (Wavefunction Inc., Irvine, CA). This computational method enabled the precise determination of molecular geometry, electronic distributions, and potential interaction sites that influence the binding affinity between the drug and DNA. Geometry optimizations were performed using the ωB97X-D range-separated hybrid functional in conjunction with the 6-311G(d,p) basis set. This functional was selected over conventional hybrids such as B3LYP because ωB97X-D includes an intrinsic empirical dispersion correction, enabling a more reliable description of non-covalent interactions. This is particularly important for ionic liquid–peptide complexes, where π–π stacking and hydrogen bonding are dominant. To account for solvent effects, the integral equation formalism polarizable continuum model with water as the solvent was employed, thereby approximating the experimental aqueous environment. The optimized geometries were subsequently validated through frequency calculations, confirming the absence of imaginary vibrational modes and ensuring that all structures correspond to true energy minima.

Following optimization, frontier molecular orbital analysis was carried out by evaluating the energies of the highest occupied molecular orbital (HOMO) and the lowest unoccupied molecular orbital (LUMO). After structural optimization, vibrational frequency analysis was conducted to verify the stability of the optimized geometries. The absence of imaginary frequencies in the vibrational spectrum confirmed that the structures corresponded to true energy minima rather than transition states or saddle points.^58–60^

Molecular electrostatic potential (MEP) mapping was conducted to visualize the electron density distribution across the drug molecule. This method allowed the identification of electrophilic and nucleophilic regions, highlighting potential interaction sites with DNA. The MEP map provided valuable insights into the electrostatic complementarity between the drug and the DNA G-quadruplex, facilitating an understanding of the molecular recognition mechanisms involved. The computational results from these DFT calculations complement experimental data, providing a molecular-level understanding of the drug's physicochemical properties, electronic features, and potential binding affinities with DNA. These insights are essential for explaining the binding interactions and stability of the drug–DNA complex, ultimately enhancing our understanding of its possible therapeutic effects.

### 2.10 Molecular docking studies for drug–G-quadruplex interactions

To clarify the molecular interactions involved in the binding of the anticancer drug 1-VAQ with G-quadruplex DNA [d-(TTAGGGT)]_4_, molecular docking simulations were performed using AutoDock 4.2. This computational method aimed to identify the preferred binding conformation, interaction energies, and molecular forces that stabilize the drug–G-quadruplex complex. The docking process used a Lamarckian Genetic Algorithm (LGA), which combines global and local search techniques to find the most energetically favorable binding sites. Visualization and analysis of the docking results were performed with AutoDock 4.2 and Discovery Studio Visualizer (Biovia, Dassault Systems), providing detailed insights into key molecular interactions, including hydrogen bonding, π–π stacking, and electrostatic interactions.^61–63^

#### 2.10.1 Preparation of ligand and G-quadruplex DNA for docking

##### 2.10.1.1 Ligand preparation

The three-dimensional (3D) structure of the anticancer drug was optimized using Density Functional Theory (DFT) at the B3LYP/6-31G(d) level prior to docking, thereby ensuring the most stable conformation. The optimized ligand structure was then processed with AutoDockTools (ADT), which added explicit hydrogen atoms to enhance the accuracy of molecular interactions. Torsional degrees of freedom were assigned to account for molecular flexibility. Gasteiger charges were computed to accurately model electrostatic interactions. The final ligand structure was saved in PDBQT format, the required input format for AutoDock docking simulations.

##### 2.10.1.2 G-quadruplex DNA preparation

The three-dimensional structure of the G-quadruplex DNA [d-(TTAGGGT)]_4_ was retrieved from the Protein Data Bank (PDB) (PDB ID: 1NP9). Preprocessing steps were performed to create an accurate docking model: water molecules and counterions were removed to prevent nonspecific interactions. Polar hydrogen atoms were added to ensure correct hydrogen bonding. Kollman charges were assigned to nucleotides for proper electrostatic calculations. The processed G-quadruplex DNA structure was saved in PDBQT format to ensure compatibility with AutoDock docking simulations.

##### 2.10.1.3 Molecular docking procedure

Molecular docking simulations were performed using AutoDock 4.2, which employs the Lamarckian Genetic Algorithm (LGA) to efficiently explore binding site preferences and predict the most favorable ligand–DNA complex conformations. A grid box was carefully defined around the guanine-rich G-tetrads and loop regions to systematically evaluate drug binding. It plays a crucial role in ligand recognition and binding stability.

Following the docking simulations, the binding results were analyzed using AutoDock and Discovery Studio Visualizer, enabling a detailed assessment of interaction modes, hydrogen bond formation, π–π stacking, and electrostatic complementarity between the drug and the G-quadruplex DNA. The binding energy values obtained offered a quantitative measure of binding affinity, providing essential insights into the stability and specificity of the drug–DNA interaction. The molecular docking results offer theoretical validation for the drug's binding affinity, preferred orientation, and key molecular interactions with the G-quadruplex DNA. These computational findings complement experimental studies, creating a comprehensive framework for understanding the molecular basis of drug binding, which is crucial for advancing the development of G-quadruplex-targeting anticancer therapies.

## 3. Result and discussion

### 3.1 Nuclear magnetic resonance (NMR)

**Fig. 1:**
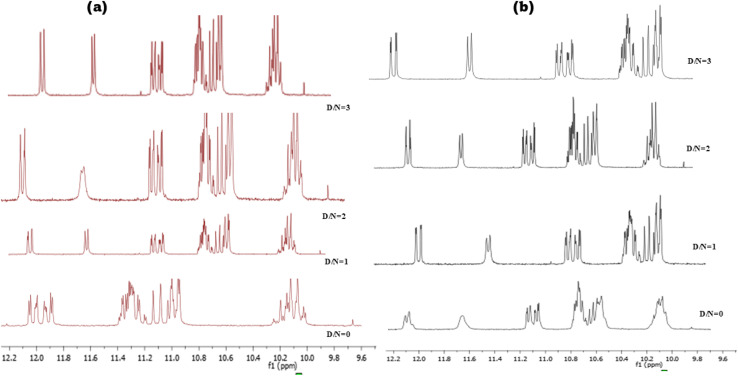
^1^H NMR titration spectra of 1-VAQ with (a) duplex DNA and (b) the G-quadruplex d-(TTAGGGT)_4_ recorded in the imino proton region (*δ* 9.6–12.2 ppm) at varying *D*/*N* ratios. Ligand addition results in concentration-dependent chemical shift perturbations, peak broadening, and intensity variations, consistent with complex formation. Comparison of the two titrations reveals substantially larger spectral perturbations for d-(TTAGGGT)_4_ than for duplex DNA, supporting preferential interaction of 1-VAQ with the G-quadruplex architecture while preserving the integrity of the hydrogen-bonded DNA structures.

To obtain molecular-level insight into the interaction of 1-VAQ with different DNA topologies, ^1^H NMR titration experiments were performed using duplex DNA and the tetramolecular G-quadruplex d-(TTAGGGT)_4_. Particular attention was focused on the imino proton region (*δ* 9.6–12.2 ppm) (Table 1), since these resonances originate from hydrogen-bonded nucleobases and are highly sensitive to ligand-induced changes in the local magnetic environment, hydrogen-bonding network, and structural organization of DNA. The ^1^H NMR spectra of duplex DNA recorded in the presence of increasing concentrations of 1-VAQ are shown in Fig. 1(a). Upon ligand addition, several imino proton resonances exhibited small but measurable chemical shift perturbations, accompanied by moderate peak broadening and intensity variations. These spectral changes indicate that the ligand interacts with duplex DNA and modifies the local electronic environment surrounding the hydrogen-bonded base pairs. However, the overall spectral pattern remained largely unchanged throughout the titration, and no extensive signal disappearance or major redistribution of resonances was observed. The persistence of the characteristic duplex imino proton signals indicates that the Watson–Crick hydrogen-bonding network remains largely preserved upon ligand binding and that the native duplex architecture is not significantly disrupted.^64^

**Table 1:** Representative chemical shift perturbations (*δ*) observed upon binding of 1-VAQ to duplex DNA and the G-quadruplex d-(TTAGGGT)_4_. Positive and negative values indicate downfield and upfield shifts, respectively

Proton resonance	Duplex DNA *δ*_free_ (ppm)	Duplex DNA *δ*_bound_ (ppm)	Δ*δ* (ppm)	G-Quadruplex *δ*_free_ (ppm)	G-Quadruplex *δ*_bound_ (ppm)	Δ*δ* (ppm)
Peak 1	12.05	12.01	−0.04	12.08	11.98	−0.10
Peak 2	11.62	11.58	−0.04	11.55	11.45	−0.10
Peak 3	10.98	10.95	−0.03	10.92	10.82	−0.10
Peak 4	10.25	10.22	−0.03	10.15	10.04	−0.11

In contrast, substantially larger spectral perturbations were observed for the G-quadruplex d-(TTAGGGT)_4_ upon addition of 1-VAQ (Fig. 1(b)). Progressive broadening of several imino proton resonances was accompanied by more pronounced changes in chemical shift and signal intensity. The greater magnitude of these perturbations suggests that the local magnetic environment of the guanine residues is more strongly influenced by ligand binding in the quadruplex than in duplex DNA. Such behaviour indicates a closer association of the ligand with the quadruplex scaffold and reflects a stronger perturbation of the hydrogen-bonded guanine tetrad environment. Importantly, although significant line broadening and chemical shift changes were observed, the characteristic imino proton resonances of the G-quadruplex remained detectable throughout the titration. This observation demonstrates that the guanine tetrad hydrogen-bonding network is retained and that the folded quadruplex structure remains intact upon ligand association. The absence of extensive signal loss or collapse of the imino proton region further indicates that ligand binding does not induce unfolding or major structural destabilization of the quadruplex framework.^65^

A direct comparison of the duplex DNA and G-quadruplex titrations clearly demonstrates that 1-VAQ produces substantially greater perturbation of the quadruplex spectrum. The larger chemical shift changes increased peak broadening, and more pronounced intensity variations observed for d-(TTAGGGT)_4_ indicate that the quadruplex environment is considerably more responsive to ligand binding than the duplex structure. These findings suggest that the G-quadruplex provides a more favorable recognition environment for 1-VAQ and are fully consistent with the significantly higher binding constants obtained from UV-visible absorption and electrochemical measurements.^66^

The observed peak broadening indicates changes in the exchange dynamics between free and bound states, while the chemical shift perturbations reflect alterations in the electronic environment surrounding hydrogen-bonded nucleobases. The more pronounced broadening observed for the G-quadruplex relative to duplex DNA suggests a greater degree of ligand-induced perturbation and stronger association with the quadruplex scaffold. Although the NMR data do not permit unambiguous assignment of a unique binding mode, they clearly demonstrate topology-dependent ligand recognition and provide direct molecular evidence that the interaction of 1-VAQ with d-(TTAGGGT)_4_ is significantly stronger than that with duplex DNA.

### 3.2 Circular dichroism (CD) spectra analysis

**Fig. 2:**
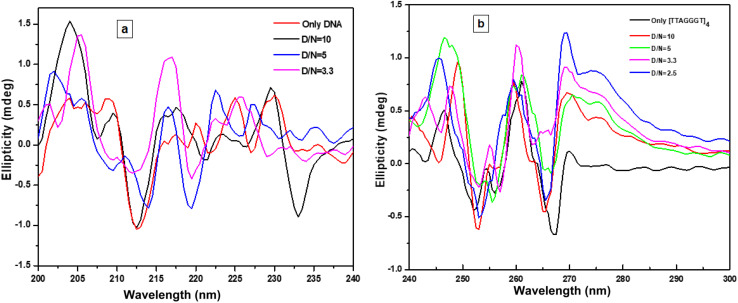
Comparative CD spectra of (a) duplex DNA and (b) G-quadruplex d-(TTAGGGT)_4_ in the presence of increasing concentrations of 1-VAQ. Duplex DNA shows characteristic bands centered at approximately 205–206 nm, 212–215 nm, and 228–230 nm, whereas the G-quadruplex exhibits characteristic bands in the 248–250 nm, 260–262 nm, and 268–278 nm regions. Ligand binding produces only modest changes in the duplex DNA spectrum but induces pronounced enhancement of the G-quadruplex bands, particularly the positive band near 270 nm, indicating stronger interaction of 1-VAQ with the G-quadruplex architecture.

Circular dichroism (CD) spectroscopy was employed to investigate the effect of 1-VAQ on the secondary structures of duplex DNA and the tetramolecular G-quadruplex d-(TTAGGGT)_4_ and to evaluate the relative selectivity of the ligand toward these two DNA topologies. Because CD signals are highly sensitive to nucleic acid conformation and base-stacking arrangements, CD spectroscopy provides valuable information on ligand-induced structural perturbations and on the preservation of topology. As shown in Fig. 2(a), the CD spectrum of duplex DNA exhibited characteristic positive bands at approximately 205–206 nm and 228–230 nm, together with negative bands at approximately 212–215 nm and 233–235 nm. Upon addition of 1-VAQ, only moderate changes in ellipticity were observed, while the overall spectral profile remained essentially unchanged. The retention of the characteristic duplex DNA bands indicates that ligand binding does not induce significant structural reorganization of the double-helical framework. The relatively small changes in ellipticity suggest limited perturbation of the duplex structure and are indicative of comparatively weaker interaction between 1-VAQ and duplex DNA.^67^

In contrast, the CD spectrum of d-(TTAGGGT)_4_ displayed a markedly different response upon ligand addition (Fig. 2(b)). The G-quadruplex exhibited characteristic positive bands at 248–250 nm, 260–262 nm, and 268–278 nm, together with negative bands near 252–255 nm and 266–268 nm. Progressive, concentration-dependent enhancement of the positive CD bands, particularly in the 268–280 nm region, was observed as G-quadruplex concentration increased. Importantly, the characteristic quadruplex spectral signature was retained throughout the titration and no significant wavelength shifts, band inversion, or loss of signal intensity were detected. These observations demonstrate that the folded G-quadruplex structure remains intact upon ligand binding and that 1-VAQ does not induce unfolding or major topological rearrangement of the quadruplex.^68^

A direct comparison of the duplex DNA and G-quadruplex titrations reveals substantially larger ligand-induced CD perturbations in the quadruplex system. While duplex DNA displayed only modest changes in ellipticity, the G-quadruplex exhibited pronounced concentration-dependent enhancement of the characteristic positive bands. The significantly greater CD response observed for d-(TTAGGGT)_4_ indicates that the interaction of 1-VAQ with the G-quadruplex is considerably stronger than that with duplex DNA under identical experimental conditions. This differential behaviour demonstrates a clear preference of the ligand for the G-quadruplex architecture.

The enhanced ellipticity observed for the G-quadruplex may be attributed to ligand-induced perturbation of the electronic environment associated with the stacked guanine tetrads while preserving the overall quadruplex fold. The larger CD response compared with duplex DNA suggests that the planar anthraquinone chromophore of 1-VAQ experiences a more favourable interaction with the G-quadruplex scaffold, which contains exposed G-tetrad surfaces and accessible external binding sites that are absent in the compact duplex helix. Consequently, the quadruplex architecture provides a more suitable binding platform for the ligand than the corresponding duplex DNA structure. Although CD spectroscopy alone cannot unambiguously distinguish between end-stacking, groove binding, or other external binding modes, the preservation of the quadruplex topology together with the pronounced concentration-dependent enhancement of the characteristic G-quadruplex bands is consistent with an external binding interaction. The precise binding mode was therefore further evaluated using complementary UV-visible absorption, fluorescence, NMR, and electrochemical studies. Although the observed spectroscopic changes clearly demonstrate interaction between 1-VAQ and d-(TTAGGGT)_4_, the available CD and NMR data do not permit unambiguous assignment of a unique binding mode. The results are consistent with an external association mechanism; however, alternative binding modes, including groove binding or other surface-associated interactions, cannot be excluded on the basis of the present data alone.

#### 3.2.1 Thermal stability studies

**Fig. 3:**
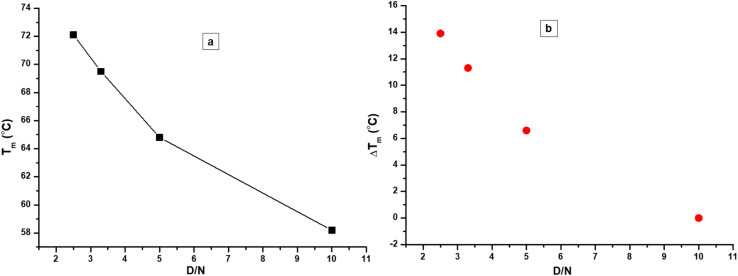
CD thermal denaturation analysis of the G-quadruplex d-(TTAGGGT)_4_ in the presence of 1-VAQ. (a) Variation of the melting temperature (*T*_m_) as a function of *D*/*N* ratio, showing a progressive increase in thermal stability with decreasing *D*/*N* ratio (increasing relative ligand concentration). (b) Corresponding changes in melting temperature (Δ*T*_m_) calculated relative to free d-(TTAGGGT)_4_. Positive (Δ*T*_m_) values ranging from +6.6 to +13.9 °C demonstrate significant ligand-induced stabilization of the G-quadruplex structure. The concentration-dependent increase in both (*T*_m_) and (Δ*T*_m_) indicates that 1-VAQ effectively enhances the thermal stability of the folded quadruplex and increases its resistance toward thermal unfolding.

The effect of 1-VAQ on the thermal stability of the G-quadruplex d-(TTAGGGT)_4_ was investigated using CD melting experiments by monitoring the characteristic quadruplex CD signal as a function of temperature. The melting temperature (*T*_m_) corresponds to the midpoint of the thermal unfolding transition and is widely used as a quantitative measure of G-quadruplex stability. An increase in (*T*_m_), upon ligand binding indicates that a higher thermal energy is required to disrupt the folded quadruplex structure, reflecting ligand-induced stabilization as shown in Fig. 3.

Upon addition of 1-VAQ, a progressive increase in the melting temperature of d-(TTAGGGT)_4_ was observed, indicating enhanced thermal stabilization of the G-quadruplex structure. The free G-quadruplex exhibited a melting temperature (*T*_m_) of 58.2 °C at a *D*/*N* ratio of 10. As the *D*/*N* ratio decreased, reflecting an increase in the relative concentration of 1-VAQ, the melting temperature increased to 64.8 °C at *D*/*N* = 5, corresponding to a (Δ*T*_m_) value of +6.6 °C. Further decreases in the *D*/*N* ratio to 3.3 and 2.5 resulted in additional stabilization, with (*T*_m_) values increasing to 69.5 °C and 72.1 °C, corresponding to (Δ*T*_m_) values of +11.3 °C and +13.9 °C, respectively. The concentration-dependent increase in (*T*_m_) demonstrates that 1-VAQ effectively stabilizes the folded G-quadruplex structure and significantly enhances its resistance toward thermal unfolding.

### 3.3 Absorption spectroscopy analysis

**Fig. 4:**
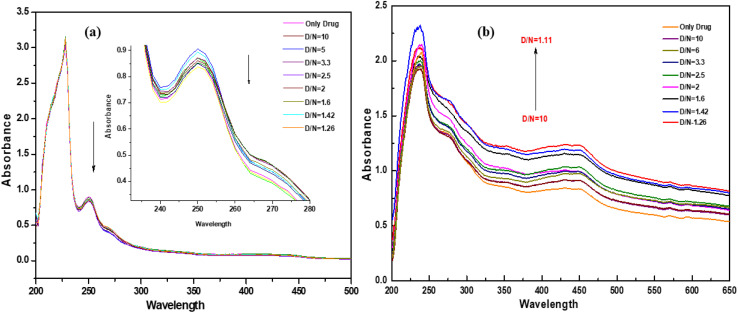
Comparative UV-Vis absorption titration spectra of 1-VAQ (20 µM) with (a) duplex DNA and (b) G-quadruplex DNA d-(TTAGGGT)_4_ recorded in phosphate buffer containing 100 mM KCl at 25 °C. Progressive addition of DNA (*D*/*N* = 10–1.26 for duplex DNA and *D*/*N* = 10–1.11 for G-quadruplex DNA) resulted in concentration-dependent spectral changes. The greater hypochromism observed for the G-quadruplex system indicates stronger electronic coupling and a higher binding affinity of 1-VAQ for G-quadruplex DNA than for duplex DNA.

The interaction of 1-VAQ with duplex DNA and the tetramolecular G-quadruplex d-(TTAGGGT)_4_ was investigated by UV-visible absorption spectroscopy to evaluate binding affinity and DNA topology selectivity. As shown in Fig. 4(a), in the duplex DNA system, a moderate hypochromic effect was observed in the 240–280 nm region, without significant shifts in the absorption maxima. The decrease in absorbance reflects perturbation of the chromophore's electronic environment upon DNA association and is commonly attributed to electronic coupling between the aromatic ligand and nucleobases. The absence of a substantial wavelength shift suggests that the interaction does not significantly alter the electronic structure of the anthraquinone chromophore.

In contrast, the G-quadruplex (TTAGGGT)_4_ (Fig. 4(b)) system exhibited much larger concentration-dependent spectral perturbations, indicating stronger interaction between 1-VAQ and d-(TTAGGGT)_4_. The pronounced changes in absorbance observed throughout the UV and visible regions suggest efficient electronic communication between the ligand and the quadruplex scaffold. The spectral response is consistent with strong π–π interactions involving the extended aromatic anthraquinone core and the guanine-rich tetrad surface.^69^

The principal absorption bands of anthraquinone derivatives arise from π–π* transitions of the conjugated aromatic system together with weaker *n*–π* transitions associated with the carbonyl groups. Upon interaction with DNA, these transitions become sensitive to changes in the local electronic environment. The observed hypochromic behavior indicates partial stabilization of the excited electronic states through stacking interactions and reduced transition probability resulting from chromophore association with DNA bases. Such effects are considerably more pronounced in the G-quadruplex system, reflecting stronger electronic coupling between the ligand and the guanine tetrads.^70^

Notably, the absence of large bathochromic shifts (>10 nm), which are often associated with deep intercalative insertion between DNA base pairs, suggests that the binding process does not involve extensive disruption of the native DNA architecture. Instead, the observed spectral behaviour is consistent with an external association mechanism in which the aromatic anthraquinone chromophore interacts with accessible nucleobase surfaces. For the G-quadruplex (TTAGGGT)_4_, the exceptionally high binding constants and pronounced spectral perturbations are consistent with association at the terminal G-tetrad surfaces through π–π stacking interactions, whereas the weaker response observed for duplex DNA suggests a less favorable binding environment. Therefore, the UV-visible absorption data indicate that binding of 1-VAQ primarily affects the electronic transitions of the anthraquinone chromophore through interaction with nucleobase π-systems. The substantially larger perturbations observed for d-(TTAGGGT)_4_ compared to duplex DNA indicate stronger electronic coupling and a significantly greater affinity for the G-quadruplex architecture.^67^

**Fig. 5:**
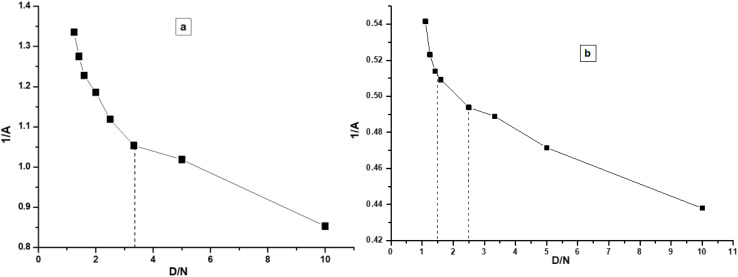
Reciprocal absorbance (1/*A*) *versus D*/*N* plots obtained from the titration of 20 µM 1-VAQ with (a) duplex DNA at 250 nm and (b) G-quadruplex DNA d-(TTAGGGT)_4_ at 481 nm. The observed inflection points at *D*/*N* ≈ 3.6 for duplex DNA and *D*/*N* ≈ 1.5 and 2.5 for G-quadruplex DNA suggest distinct binding stoichiometries and preferential interaction of 1-VAQ with the G-quadruplex.

The reciprocal absorbance plot (1/*A versus D*/*N*) shown in Fig. 5 at 280 nm and 481 nm revealed distinctly different binding behaviours for duplex DNA and d-(TTAGGGT)_4_. In the duplex DNA system, a single inflection points at *D*/*N* = 3.6, was observed, indicating the presence of one dominant binding event between 1-VAQ and the DNA structure. In contrast, the G-quadruplex exhibited two distinct inflection points at *D*/*N* = 1.5 and 2.5, suggesting the occurrence of two energetically distinguishable binding events, as illustrated in Fig. 5. The first inflection point (*D*/*N* = 1.5) corresponds to a higher-affinity interaction, while the second inflection point (*D*/*N* = 2.5) indicates the presence of an additional binding event occurring at higher DNA concentrations. The presence of two inflection points is consistent with multiple binding environments or binding sites within the G-quadruplex structure.

**Fig. 6:**
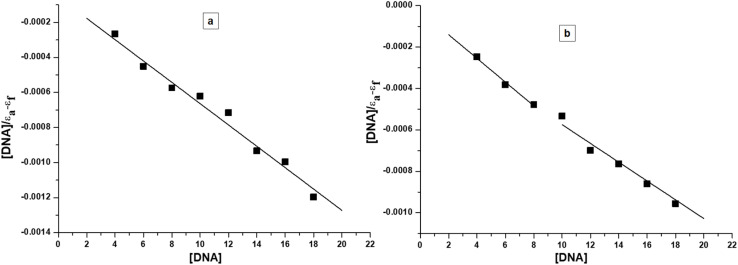
Benesi–Hildebrand plots for the interaction of 1-VAQ with (a) duplex DNA and (b) G-quadruplex DNA d-(TTAGGGT)_4_. The plots of [DNA]/(*ε*_a_ − *ε*_f_) *versus* ([DNA]) were constructed from UV-Vis absorption titration data and used to determine the intrinsic binding constant (*K*_b_) of the ligand–DNA complexes. The strong linear correlation observed in both systems supports the applicability of the binding model, and the comparison of the plots indicates a stronger affinity of 1-VAQ for G-quadruplex DNA than for duplex DNA.

Binding constants (*K*_b_) calculated from the absorption titration data further support the preferential recognition of the G-quadruplex by 1-VAQ. The duplex DNA system exhibited a binding constant (*K*_b_) of 1.09 × 10^4^ M^−1^, indicative of moderate ligand–DNA association (Fig. 6(a)). In contrast, analysis of the G-quadruplex titration revealed two distinct binding events with binding constants (*K*_b_) of 1.07 × 10^6^ M^−1^ and 2.13 × 10^6^ M^−1^ (Fig. 6(b)). The occurrence of two binding constants suggests the presence of multiple energetically distinguishable binding events within the G-quadruplex system. Such behaviour is consistent with the availability of more than one accessible binding site on the tetramolecular quadruplex, most likely associated with the terminal G-tetrad surfaces. The exceptionally high binding constants indicate that these interactions are substantially more favorable than those occurring with duplex DNA.

The thermodynamic favorability of the binding process was further evaluated using the standard Gibbs free energy (Δ*G*). The calculated (Δ*G*) value for duplex DNA was found to be (−23.06) kJ mol^−1^, whereas significantly more negative values of (−34.40) and (−36.12) kJ mol^−1^ were obtained for the two G-quadruplex d-(TTAGGGT)_4_ binding events. The negative free energy values confirm that the interaction is spontaneous in both systems. However, the substantially more negative (Δ*G*) values observed for d-(TTAGGGT)_4_ indicate a thermodynamically more favorable interaction and further support the stronger affinity of 1-VAQ toward the quadruplex structure. A direct comparison of the duplex DNA and G-quadruplex d-(TTAGGGT)_4_ titrations clearly demonstrates that 1-VAQ interacts much more strongly with d-(TTAGGGT)_4_ than with duplex DNA. While duplex DNA exhibits only modest spectral perturbations and moderate binding affinity, the G-quadruplex displays pronounced concentration-dependent absorbance changes together with exceptionally high binding constants and highly favorable Gibbs free energy values.

Although UV-visible absorption spectroscopy alone cannot unambiguously establish the exact binding mode, the large spectral perturbations, exceptionally high binding constants, and thermodynamically favorable interaction observed for d-(TTAGGGT)_4_, together with the complementary circular dichroism, NMR results, strongly support preferential association of 1-VAQ with the G-quadruplex structure. Collectively, these findings demonstrate that the quadruplex architecture provides a significantly more favorable binding platform for 1-VAQ than the corresponding duplex DNA structure.

### 3.4 Fluorescence titrations

Fluorescence spectroscopy was employed to further investigate the interaction of 1-VAQ with duplex DNA and the G-quadruplex d-(TTAGGGT)_4_ and to evaluate the influence of DNA topology on the photophysical behaviour of the ligand. The fluorescence emission spectra revealed markedly different responses between the two DNA systems, indicating that the ligand's local environment is strongly dependent on DNA architecture.

**Fig. 7:**
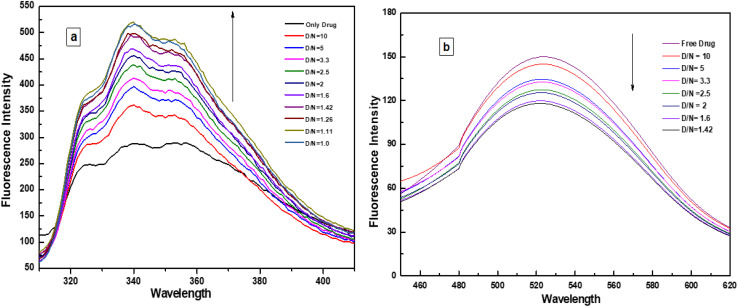
Fluorescence titration spectra of 20 µM 1-VAQ with (a) duplex DNA and (b) G-quadruplex DNA d-(TTAGGGT)_4_ recorded in 10 mM phosphate buffer containing 100 mM KCl at 25 °C. Upon increasing DNA concentration, duplex DNA produced a marked enhancement in fluorescence intensity, suggesting restriction of non-radiative relaxation pathways upon binding. In contrast, G-quadruplex DNA induced significant fluorescence quenching, consistent with strong π–π stacking interactions and possible photoinduced electron transfer between the anthraquinone chromophore and guanine tetrads.

As shown in Fig. 7(a), gradual addition of duplex DNA to 1-VAQ resulted in a progressive enhancement of fluorescence intensity throughout the titration. The emission maximum remained essentially unchanged at approximately 340–350 nm, indicating that ligand binding does not significantly alter the intrinsic electronic transitions of the anthraquinone chromophore. The observed increase in fluorescence intensity suggests that association with duplex DNA places the ligand in a more restricted environment, reducing non-radiative relaxation processes and increasing fluorescence efficiency. The absence of fluorescence quenching indicates that duplex DNA does not substantially promote the ligand's excited-state deactivation.^71^

In contrast, titration of 1-VAQ with the G-quadruplex d-(TTAGGGT)_4_ produced a concentration-dependent decrease in fluorescence intensity (Fig. 7(b)). The free ligand exhibited the highest emission intensity, whereas the progressive addition of quadruplex DNA resulted in a systematic decrease in fluorescence intensity. Despite significant intensity changes, no appreciable shift in the emission maximum was observed, indicating that the primary effect of quadruplex binding is on excited-state relaxation pathways rather than on the fluorophore's fundamental electronic structure. The pronounced quenching behaviour demonstrates that interaction with d-(TTAGGGT)_4_ perturbs the excited-state properties of the ligand considerably more than interaction with duplex DNA.^72^

**Fig. 8:**
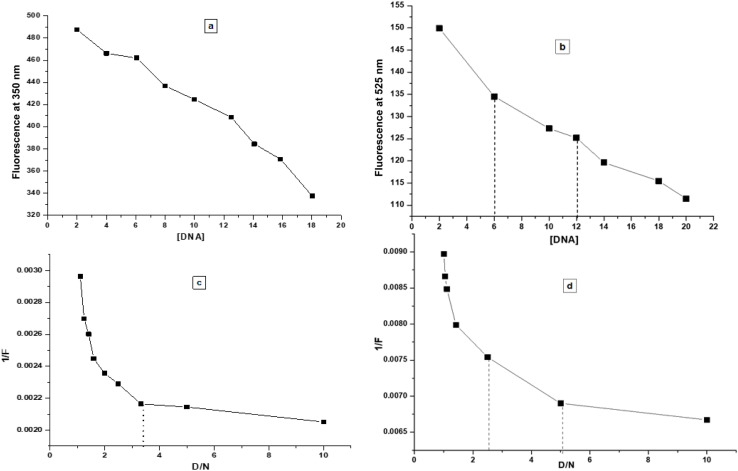
Fluorescence studies of the interaction of 1-VAQ with duplex DNA and the G-quadruplex d-(TTAGGGT)_4_. (a) Fluorescence emission spectra of 20 µM 1-VAQ upon titration with increasing concentrations of duplex DNA at different *D*/*N* ratios, showing progressive enhancement of fluorescence intensity at 350 nm. (b) Fluorescence emission spectra of 20 µM 1-VAQ upon titration with increasing concentrations of d-(TTAGGGT)_4_ at different *D*/*N* ratios, showing concentration-dependent fluorescence quenching at 505 nm (c) A graph illustrating the reciprocal of fluorescence intensity (1/*F*) at 350 nm plotted against the *D*/*N* ratio for duplex DNA with characteristic inflection point at *D*/*N* = 3.6 (d) a graph illustrating the reciprocal of fluorescence intensity (1/*F*) at 505 nm plotted against the *D*/*N* ratio, for d-(TTAGGGT)_4_ with characteristic inflection points at *D*/*N* = 2.4 and 4.6.

A direct comparison of the two fluorescence titrations reveals fundamentally different photophysical responses. Whereas duplex DNA induces fluorescence enhancement, the G-quadruplex promotes fluorescence quenching. These contrasting behaviours indicate that 1-VAQ experiences distinct microenvironments upon association with the two DNA topologies. The stronger perturbation of fluorescence observed in the presence of d-(TTAGGGT)_4_ suggests a more effective interaction between the ligand and the quadruplex structure. A closer examination of the emission spectra further highlights the distinct behaviour of the two DNA systems. In the duplex DNA titration, the fluorescence intensity increased progressively with increasing DNA concentration, reaching a maximum enhancement at the lowest *D*/*N* ratios (Fig. 8). The overall shape of the emission band remained essentially unchanged, and no significant shift in the emission maximum was observed. This behaviour indicates that the ligand maintains a similar emissive state upon binding while experiencing an environment that favors radiative decay processes. In contrast, the G-quadruplex titration exhibited a systematic decrease in fluorescence intensity over the entire emission profile. The quenching effect became progressively more pronounced with increasing quadruplex concentration, demonstrating a clear concentration–dependent interaction. Notably, the emission maximum remained nearly constant throughout the titration, indicating that the observed spectral changes arise primarily from variations in fluorescence intensity rather than major alterations in the electronic transitions of the fluorophore.^73^

The preservation of the spectral profile together with the pronounced decrease in emission intensity suggests that the excited-state behaviour of 1-VAQ is more strongly influenced by the G-quadruplex environment than by duplex DNA. The fluorescence results are fully consistent with the UV-visible absorption, circular dichroism, NMR, and electrochemical studies, which collectively demonstrate stronger association of 1-VAQ with d-(TTAGGGT)_4_ than with duplex DNA. Although fluorescence spectroscopy alone cannot establish the precise binding mode, the marked difference between the duplex DNA and G-quadruplex responses provides strong evidence that the quadruplex architecture offers a significantly more favorable binding environment for 1-VAQ. Collectively, these findings support the preferential recognition of the G-quadruplex structure over duplex DNA by the ligand.

The fluorescence titration experiments revealed distinctly different binding behaviour of 1-VAQ toward duplex DNA and the G-quadruplex d-(TTAGGGT)_4_. In both systems, progressive changes in fluorescence intensity were observed upon increasing DNA concentration, indicating the formation of ligand–DNA complexes. At higher DNA concentrations, the fluorescence response approached saturation, suggesting progressive occupation of the available ligand-binding environments (Fig. 8(a and b)). The corresponding reciprocal plots (1/*F versus D*/*N*) exhibited clear inflection points (Fig. 8(c and d)), supporting the occurrence of discrete binding events during complex formation.

**Fig. 9:**
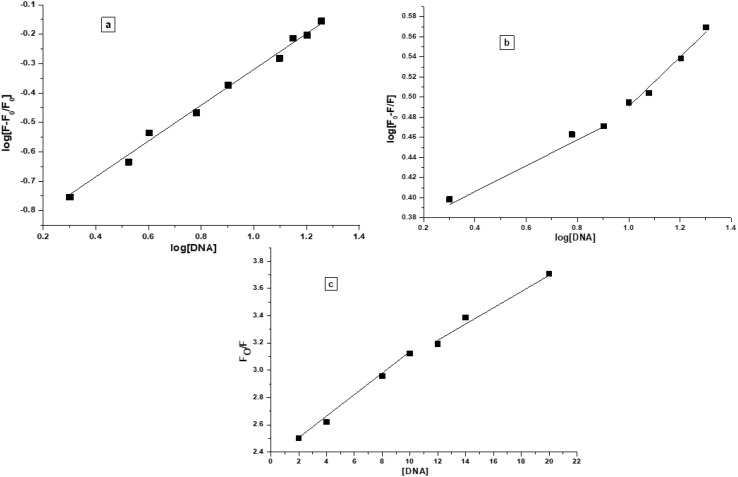
Fluorescence binding analysis of 1-VAQ with duplex DNA and the G-quadruplex d-(TTAGGGT)_4_. (a) Double-logarithmic binding plot of (log[(*F* − *F*_0_)/*F*_0_]) *versus* (log[DNA]) at 350 nm for duplex DNA, used for determination of the binding constant (*K*_b_) (b) double-logarithmic binding plot of (log[(*F*_0_ − *F*)/*F*]) *versus* (log[DNA]) at 525 nm for the G-quadruplex d-(TTAGGGT)_4_, used for determination of the binding constant (*K*_b_) (c) Stern–Volmer plot of (*F*_0_/*F*) *versus* [DNA] at 525 nm for d-(TTAGGGT)_4_, used to determine the Stern–Volmer quenching constant (*K*_SV_). The distinct fluorescence responses observed for duplex DNA and G-quadruplex DNA reflect topology-dependent interactions of 1-VAQ with the two DNA architectures.

Quantitative analysis of the fluorescence titration data was performed using double-logarithmic binding plots of log[(*F* − *F*_0_)/*F*_0_] *versus* log[DNA] for duplex DNA and the corresponding binding model for the G-quadruplex system. The calculated binding constant for duplex DNA was found to be (*K*_b_) = 1.52 × 10^4^ M^−1^, indicating moderate ligand–DNA association. In contrast, the G-quadruplex d-(TTAGGGT)_4_ exhibited significantly higher binding constants of (*K*_b_) = 1.38 × 10^6^ M^−1^ and (*K*_b_) = 2.13 × 10^6^ M^−1^, corresponding to two distinguishable binding events (Fig. 9(a and b)). The thermodynamic favorability of the interaction was further evaluated using the standard Gibbs free energy (Δ*G*). The duplex DNA complex exhibited a (Δ*G*) value of approximately (−23.9) kJ mol^−1^, whereas substantially more negative values of (−35.0) and (−36.1) kJ mol^−1^ were obtained for the two G-quadruplex binding events. The negative free energy values confirm that ligand binding is spontaneous in both systems. However, the significantly more negative (Δ*G*) values observed for d-(TTAGGGT)_4_ indicate a considerably more favorable interaction and greater thermodynamic stability of the G-quadruplex complexes than those of duplex DNA.

Further insight into the fluorescence response of the quadruplex system was obtained from Stern–Volmer analysis. The plots of (*F*_0_/*F*) *versus* [DNA] (Fig. 9(c)) yielded quenching constants (*K*_SV_) of 2.19 × 10^6^ M^−1^, for the *D*/*N* range of 10–4.6 and 2.29 × 10^6^ M^−1^, for the *D*/*N* range of 2.4–1.1. The large Stern–Volmer constants indicate highly efficient quenching of the excited state of 1-VAQ in the presence of d-(TTAGGGT)_4_ and are consistent with the formation of stable ligand–quadruplex complexes. The existence of two Stern–Volmer regions further supports the presence of multiple binding environments or binding events within the quadruplex system. A direct comparison of the fluorescence-derived binding parameters clearly demonstrates the preferential recognition of d-(TTAGGGT)_4_ by 1-VAQ. The approximately two orders of magnitude increase in binding affinity, together with the substantially more favorable Gibbs free energy values, indicates that the G-quadruplex architecture provides a significantly more favorable binding environment than duplex DNA. These findings are fully consistent with UV-visible absorption, circular dichroism, NMR, and electrochemical studies, all of which independently demonstrate a stronger interaction between 1-VAQ and the G-quadruplex structure. Collectively, the fluorescence results provide strong quantitative evidence for the selective and thermodynamically favorable association of 1-VAQ with d-(TTAGGGT)_4_ relative to duplex DNA.

### 3.5 Cyclic voltammetry (CV) analysis

**Fig. 10:**
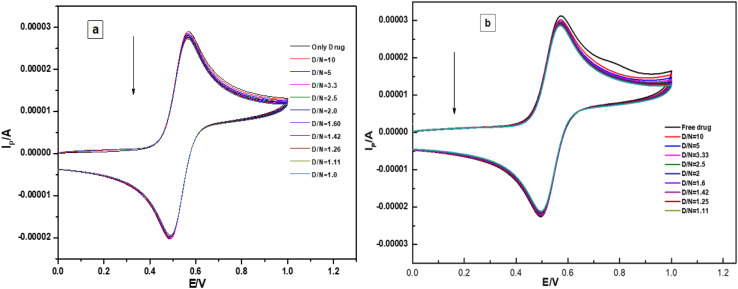
Cyclic voltammogram of 20 µM 1-VAQ with increasing concentrations of (a) duplex DNA (b) [d-(TTAGGGT)_4_] quadruplex, measured in a 10 mM TEMPO (2,2,6,6-tetramethylpiperidin-1-oxyl) and KNO_3_ buffer solution.

The interaction of 1-VAQ with duplex DNA and the tetramolecular G-quadruplex d-(TTAGGGT)_4_ was further investigated by cyclic voltammetry (CV) to evaluate the influence of DNA binding on the ligand's electrochemical behavior and to compare its affinity toward different DNA topologies. CV is a highly sensitive technique for probing DNA–ligand interactions because complex formation generally results in changes in peak current and diffusion characteristics of the electroactive species. As shown in Fig. 10(a), gradual addition of duplex DNA to 1-VAQ resulted in a progressive decrease in both anodic and cathodic peak currents. The reduction in peak current is attributed to the formation of a DNA–ligand complex with a larger hydrodynamic volume and, consequently, a lower diffusion coefficient than the free ligand. Notably, only minor changes in peak potential were observed throughout the titration, indicating that the redox process of 1-VAQ remains essentially unchanged and that the interaction primarily affects the diffusion properties of the ligand. The relatively modest decrease in current suggests a moderate interaction between 1-VAQ and duplex DNA.^69,70^

In contrast, titration of 1-VAQ with d-(TTAGGGT)_4_ produced a substantially greater attenuation of both oxidation and reduction peak currents under identical experimental conditions (Fig. 10(b)). The pronounced decrease in current intensity reflects the formation of a more strongly associated ligand–quadruplex complex with reduced mobility in solution. Similar to duplex DNA, no significant shift in peak potential was observed, indicating that the redox center of the ligand remains electrochemically accessible and that the interaction does not significantly alter the electron-transfer mechanism. However, the markedly larger current suppression observed in the presence of the G-quadruplex clearly demonstrates a stronger interaction with d-(TTAGGGT)_4_ than with duplex DNA.^74,75^

**Fig. 11:**
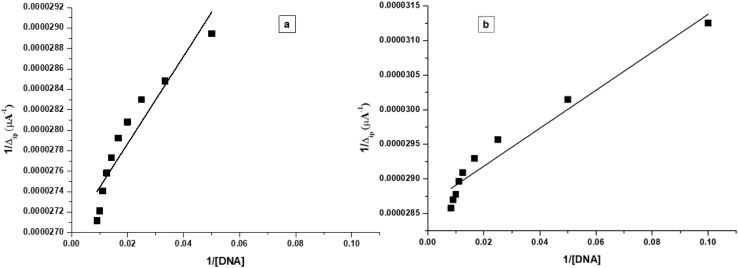
Graphical representation of 1/Δ*I*_p_*versus* 1/[DNA], illustrating the binding constant (*K*_b_) (a) for duplex DNA, (b) for G-quadruplex [d-(TTAGGGT)_4_], calculated over a *D*/*N* range of 10–1.1.

The binding constants (*K*_b_) calculated from the electrochemical data were found to be 1.504× 10^4^ for duplex DNA and 2.87 × 10^5^ for d-(TTAGGGT)_4_ (Fig. 11). The approximately nineteen-fold higher binding constant obtained for the G-quadruplex unequivocally demonstrates the preferential recognition of the quadruplex topology by 1-VAQ. This substantial difference in binding affinity indicates that the structural features of the G-quadruplex provide a considerably more favorable binding environment than those of the corresponding duplex DNA. The thermodynamic favorability of the binding process was further evaluated using the standard Gibbs free energy change (Δ*G*). The calculated (Δ*G*) values were (−23.82) kJ mol^−1^ for duplex DNA and (−31.14) kJ mol^−1^ for d-(TTAGGGT)_4_. The negative free energy values confirm that the binding process is spontaneous in both systems. Importantly, the significantly more negative (Δ*G*) value obtained for the G-quadruplex complex indicates a thermodynamically more favorable interaction and further supports the higher affinity of 1-VAQ toward d-(TTAGGGT)_4_.

A direct comparison of the electrochemical responses of the two DNA systems clearly reveals that the G-quadruplex induces a much greater reduction in peak current and exhibits a substantially larger binding constant than duplex DNA. These findings are fully consistent with UV-visible absorption, fluorescence, circular dichroism, and NMR studies, all of which demonstrate a stronger interaction between 1-VAQ and the G-quadruplex structure. Collectively, the electrochemical results provide compelling evidence that 1-VAQ preferentially recognizes and binds the G-quadruplex d-(TTAGGGT)_4_ over duplex DNA. The absence of major peak potential shifts together with the preservation of the redox characteristics of the ligand suggests that the interaction occurs predominantly through an external binding mode rather than through a mechanism that significantly perturbs the electronic structure of the redox-active anthraquinone core. Therefore, the CV results support a strong, selective association of 1-VAQ with the G-quadruplex architecture while preserving the ligand's intrinsic electrochemical properties.

Although the binding constants obtained from UV-visible absorption, fluorescence spectroscopy, and cyclic voltammetry differ quantitatively, such variations are commonly observed because each technique probes a different aspect of the ligand–DNA interaction. UV-visible absorption and fluorescence measurements monitor changes in the spectroscopic properties of the ligand in bulk solution, whereas cyclic voltammetry reflects alterations in electrochemical behaviour, diffusion characteristics, and electron-transfer processes at the electrode interface. Consequently, the electrochemically derived binding constants may differ from those obtained spectroscopically. Nevertheless, all three techniques consistently indicate strong interaction of 1-VAQ with d-(TTAGGGT)_4_ and demonstrate substantially greater affinity for the G-quadruplex than for duplex DNA.

A comparison of the binding parameters obtained from UV-visible absorption, fluorescence, and electrochemical measurements listed in Table 2, clearly demonstrates the preferential interaction of 1-VAQ with the G-quadruplex d-(TTAGGGT)_4_ relative to duplex DNA. Across all three experimental techniques, the binding constants (*K*_b_) obtained for the G-quadruplex are approximately one to two orders of magnitude higher than those measured for duplex DNA. Correspondingly, the Gibbs free energy values for the G-quadruplex complexes are substantially more negative (−31) to (−37) kJ mol^−1^ than those obtained for duplex DNA (−23) to (−24) kJ mol^−1^. The consistently higher binding constants and more favorable free energy values indicate that the interaction of 1-VAQ with d-(TTAGGGT)_4_ is both thermodynamically more favorable and significantly stronger than its interaction with duplex DNA.

**Table 2:** Comparison of binding constants (*K*_b_) and Gibbs free energy values (Δ*G*) for the interaction of 1-VAQ with duplex DNA and the G-quadruplex d-(TTAGGGT)_4_ determined using different experimental techniques

Techniques	Binding constant (*K*_b_) for duplex DNA	Gibbs free energy (Δ*G*) for duplex DNA kJ mol^−1^	Binding constant (*K*_b_) for G-quadruplex [d-(TTAGGGT)_4_]	Gibbs free energy (Δ*G*) for G-quadruplex [d-(TTAGGGT)_4_] kJ mol^−1^
UV-visible spectroscopy	1.09 × 10^4^ M^−1^	−23.06	1.07 × 10^6^ M^−1^ at *D*/*N* = 10–2.5	−34.4
			3.16 × 10^6^ M^−1^ at *D*/*N* = 2–1.1	−37.0
Fluorescence spectroscopy	1.52 × 10^4^ M^−1^	−23.90	1.38× 10^6^ M^−1^ at *D*/*N* = 10–2.5	−35.0
			2.13× 10^6^ M^−1^ at *D*/*N* = 2–1.1	−36.1
Cyclic voltammetry	1.58 × 10^4^ M^−1^	−23.82	*K*_b_ = 2.87× 10^5^ M^−1^ at *D*/*N* = 10–1.1	−31.1

### 3.6 Dynamic light scattering for drug–G-quadruplex interactions

**Fig. 12:**
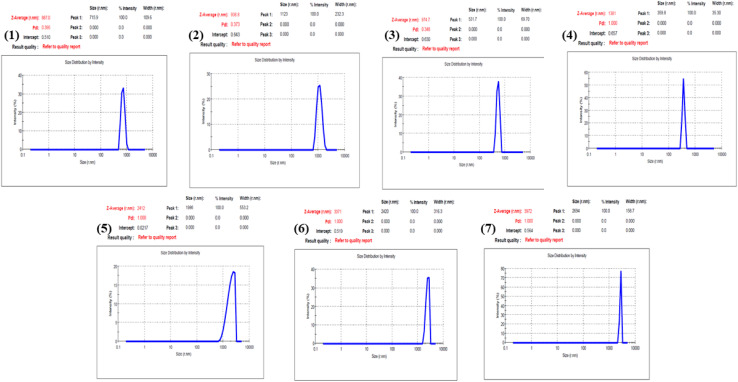
Dynamic light scattering of 20 µM 1-VAQ with increasing concentrations of d-[TTAGGGT]_4_, showing changes in *Z*-average hydrodynamic size and polydispersity index (PDI).

Dynamic light scattering (DLS) analysis was performed to investigate the hydrodynamic behavior of 1-VAQ and its assemblies with d-[TTAGGGT]_4_ in solution over a range of drug-to-nucleotide ratios. The free 1-VAQ solution exhibited a *Z*-average hydrodynamic diameter of 887.3 nm with a PDI value of 0.395, indicating the presence of moderately polydisperse species in aqueous medium. Upon progressive addition of d-[TTAGGGT]_4_, the measured hydrodynamic diameter increased systematically from 938.8 to 3972 nm, accompanied by increasing PDI values (Table 3 and Fig. 12), demonstrating concentration-dependent association behavior between the ligand and the G-quadruplex system.^76^ The relatively large hydrodynamic diameters observed in this study are considerably greater than the dimensions expected for an individual ligand–DNA complex and therefore likely reflect the formation of higher-order supramolecular assemblies in solution rather than discrete monomeric complexes alone.^77^ In DLS measurements, the reported hydrodynamic diameter corresponds to the effective solvated radius of dynamically diffusing particles and is highly sensitive to intermolecular clustering, hydration shell effects, and transient associative networks in solution. Consequently, planar aromatic ligands capable of strong intermolecular π–π interactions may generate substantially larger apparent particle sizes than the actual molecular dimensions of the primary binding complex.

**Table 3:** Variation in *Z*-average hydrodynamic size and PDI values for the drug–G-quadruplex d-[TTAGGGT]_4_ complexes highlights changes in particle size distribution and sample uniformity upon drug addition binding

Sr no.	Condition	*Z*-Average size (nm)	PDI
1	Drug alone	887.3	0.395
2	Drug + d-[TTAGGGT]_4_ (low)	938.8	0.573
3	Drug + d-[TTAGGGT]_4_ (moderate)	974.7	0.348
4	Drug + d-[TTAGGGT]_4_ (higher)	1381	1
5	Drug + d-[TTAGGGT]_4_ (higher)	2412	1
6	Drug + d-[TTAGGGT]_4_ (highest)	3371	1
7	Drug + d-[TTAGGGT]_4_ (highest)	3972	1

In the present system, the extended aromatic anthraquinone scaffold of 1-VAQ, together with the exposed terminal G-quartet surfaces of d-[TTAGGGT]_4_, likely promotes secondary intermolecular association at elevated concentrations, resulting in the formation of loosely organized supramolecular ligand–G4 assemblies. The progressive increase in hydrodynamic size observed here, therefore, most likely reflects the evolution of these larger dynamic assemblies rather than irreversible precipitation. At lower DNA concentrations, moderate PDI values indicate relatively dispersed populations of ligand–DNA assemblies, whereas the transition to PDI values approaching 1.000 at higher *D*/*N* ratios suggests the coexistence of multiple assembly populations with broad size distributions.^78^ Importantly, despite this increased heterogeneity, no visible precipitation or macroscopic instability was observed during measurements. Additionally, the UV-Vis spectra did not exhibit severe baseline distortion due to scattering at higher wavelengths, indicating that the system remained colloidally dispersed under the experimental conditions.^79^

Importantly, the DLS data are interpreted here as complementary evidence for concentration-dependent supramolecular association in solution rather than as a direct determination of discrete molecular binding stoichiometry. The molecular-level binding mode is instead established through the combined spectroscopic, NMR, electrochemical, and computational analyses. In particular, the preservation of the parallel G-quadruplex CD signature, the progressive aromatic proton perturbations observed in ^1^H NMR spectra, the hypochromic UV-Vis response, fluorescence quenching behavior, and docking-derived end-stacking geometry collectively support predominant external π–π end-stacking interactions between 1-VAQ and d-[TTAGGGT]_4_. Thus, the DLS results are consistent with a model in which primary ligand–G4 binding is accompanied by the formation of larger dynamic supramolecular assemblies in solution at elevated concentrations.

### 3.7 Density functional theory (DFT) analysis and molecular interaction analysis

**Fig. 13:**
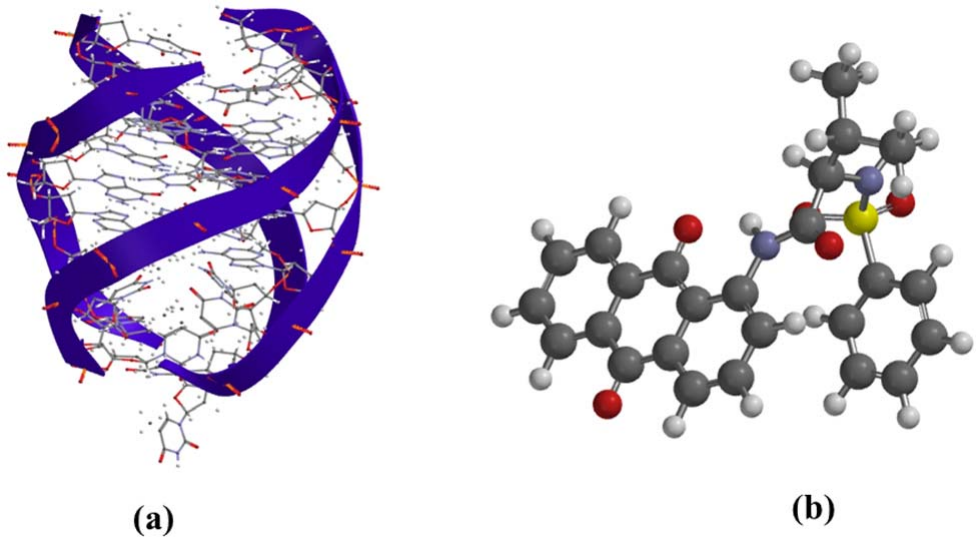
Density functional theory (DFT)-optimized molecular structures (a) optimized conformations of the G-quadruplex DNA sequence d-[TTAGGGT]_4_ (PDB ID: 1NP9), illustrating the characteristic guanine tetrad stacking and loop arrangements critical for ligand binding. (b) Anticancer ligand 1-VAQ, highlighting its planar aromatic core and functional groups relevant for interaction.

Density Functional Theory (DFT) calculations were performed to elucidate the structural and electronic basis of the interaction between 1-VAQ and the G-quadruplex DNA sequence d-[TTAGGGT]_4_. Geometry optimizations were conducted to identify the most stable configurations of both the ligand and its complex with G-quadruplex DNA, revealing key molecular features that define the interaction at atomic resolution (Fig. 13).^80,81^

#### 3.7.1 Optimized structure and binding geometry

The DFT-optimized structure reveals that the anthraquinone group lies parallel to the terminal G-tetrad surface, consistent with π–π stacking interactions previously suggested by UV-Vis and fluorescence data. The benzenesulfonamide and amide groups are directed toward the groove regions, where they form hydrogen bonds with guanine bases and the phosphate backbone, thereby enhancing the stability of the complex. This arrangement enables optimal overlap between the π-system of the anthracene core and the guanine quartets, facilitating the delocalization of electronic density across the binding interface. The calculated stabilization energy indicates strong, spontaneous binding, in line with the experimentally observed negative Gibbs free energies.^82,83^

#### 3.7.2 Electrostatic potential mapping and orbital interactions

**Fig. 14:**
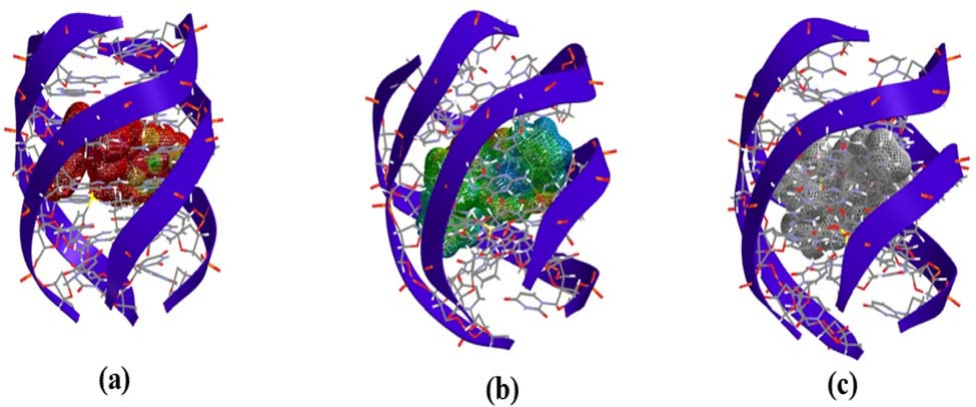
Drug–DNA binding interactions visualized through molecular electrostatic potential (MEP) and electrostatic potential (ESP) maps for the complex between 1-VAQ and DNA d-[TTAGGGT]_4_. (a) The initial binding conformation highlights MEP distribution; (b) the intermediate binding state shows ESP characteristics and molecular orbital overlap; (c) the final drug–DNA complex features enhanced ESP visualization. The electrostatic potential is color-coded from red (electron-rich) to blue (electron-deficient).

The electrostatic potential (ESP) map of the optimized complex (Fig. 14(a)) displays the charge distribution across the drug–DNA interface. Electron-rich areas (red) are concentrated around the guanine oxygen atoms, while electron-deficient areas (blue) are found on the anthraquinone carbonyl groups and sulfonamide nitrogen, enabling complementary electrostatic pairing (Fig. 14(b)). The alignment indicates efficient charge redistribution upon binding, which enhances π–π and hydrogen-bonding interactions. The molecular electrostatic potential (MEP) surface (Fig. 14(c)) illustrates these effects, with intense blue regions near the drug's cationic centers interacting with the negatively charged phosphate groups of DNA. At the same time, green areas represent moderately polar hydrogen-bonding regions. The balanced distribution of positive and negative potentials over the binding interface demonstrates electrostatic complementarity and confirms the formation of a thermodynamically favorable complex.^84,85^

### 3.8 Molecular docking analysis of drug interaction with G-quadruplex d-[TTAGGGT]_4_ DNA: insights into binding mechanism, stability, and affinity

**Fig. 15:**
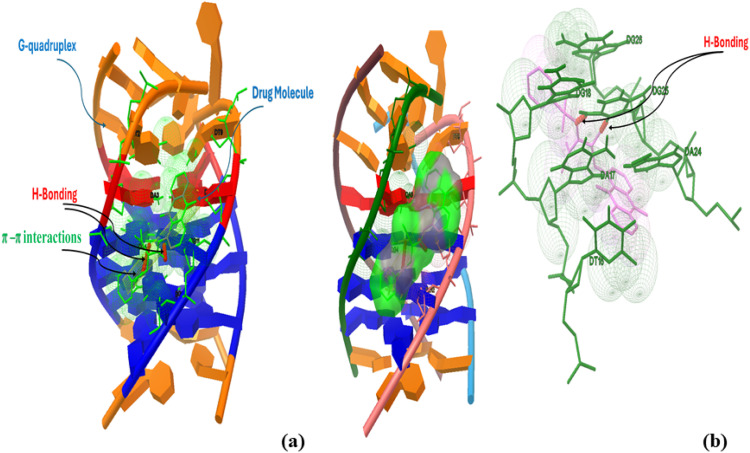
Molecular docking analysis of 1-VAQ interacting with the G-quadruplex DNA sequence d-[TTAGGGT]_4_, performed using AutoDock software. (a) A comprehensive view of the docking model illustrates the drug molecule's initial binding orientation and key π–π stacking interactions and H-bonding (indicated by black arrows) between the ligand's aromatic core and the guanine tetrads. (b) Magnified representation highlights hydrogen bonding (H-bonding, red dashed lines) between the ligands.

Molecular docking simulations were performed to determine the binding mode, affinity, and stability of 1-VAQ in complex with G-quadruplex DNA (d-[TTAGGGT]_4_), providing atomic-level insight into its stabilization mechanism (Fig. 15(a) and (b)).

#### 3.8.1 Binding mode and interaction network

Docking results showed that the drug preferentially binds to the terminal G-tetrads of the quadruplex through π–π stacking interactions between its planar anthraquinone core and guanine bases. These stacking contacts arise from delocalized π-electron overlap and serve as a primary stabilizing force, aligning with experimental UV-Vis and fluorescence findings that support π–π stacking-driven complexation. Such interactions are well known for stabilizing G-quadruplex structures by preventing strand dissociation and facilitating telomerase inhibition. Besides stacking, hydrogen bonding plays a key role in ligand binding specificity and orientation. Multiple hydrogen bonds were observed between the oxygen and nitrogen atoms of the drug and the guanine residues or phosphate groups in the G-quadruplex backbone (Fig. 15a and b). The H-bond lengths, ranging from 1.28 to 2.14 Å, are within the typical range for moderate-to-strong hydrogen bonds, indicating stable interactions that could prolong the drug's residence time on the quadruplex and improve its biological effect half-life.^86^

#### 3.8.2 Electrostatic and hydrophobic interactions

Electrostatic complementarity also supports binding, as the negatively charged DNA phosphate backbone interacts with the drug's polar and positively charged functional groups, thereby stabilizing charges and enhancing binding affinity. Additionally, hydrophobic interactions between the anthracene ring system and nonpolar pockets of the G-quadruplex help reduce desolvation energy and enhance conformational stability. These interactions collectively ensure a snug, complementary fit of the ligand within the DNA grooves.

#### 3.8.3 Binding energetics and inhibition parameters

Thermodynamic parameters derived from docking simulations indicate favourable and spontaneous binding. The calculated binding free energies (Δ*G*) for various poses range from −8.52 to −8.84 kcal mol^−1^, confirming strong and energetically favourable interactions. The lowest-energy configuration (Δ*G* = −8.84 kcal mol^−1^) corresponds to the most stable binding mode, dominated by π–π stacking and hydrogen bonding. The associated inhibition constants (*K*_i_) vary between 341.59 nM and 5.07 µM, with the lowest *K*_i_ value signifying nanomolar-level affinity, indicative of potent G-quadruplex binding. Docking energy decomposition showed that π–π stacking and hydrogen bonding contributed the most to stabilization, followed by van der Waals and electrostatic forces. The energetically optimized binding conformation suggests that increasing the planarity of the aromatic core or adding more electron-donating groups could further strengthen π–π stacking, providing valuable guidance for future structural improvements and optimization.^87^

#### 3.8.4 Structural visualization and complementarity

**Fig. 16:**
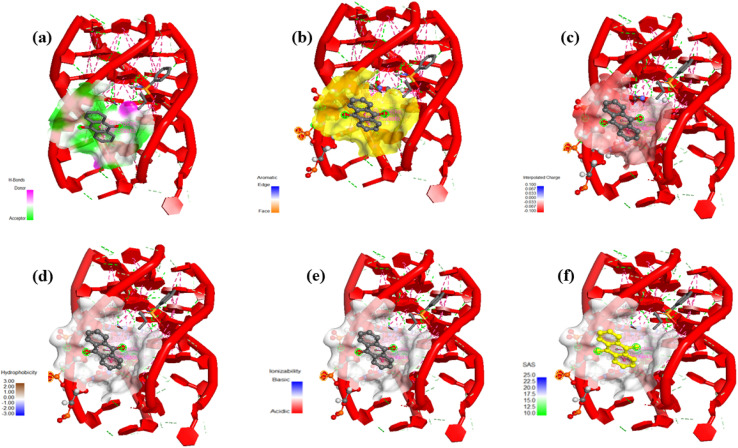
Molecular interaction surfaces of the anticancer ligand 1-VAQ in complex with the G-quadruplex DNA sequence d-[TTAGGGT]_4_, visualized using Discovery Studio. (a) Hydrogen bond donor and acceptor regions, highlighting key interaction sites within the binding interface. (b) Electrostatic potential mapped onto the molecular surface, indicating charge complementarity between the DNA and ligand. (c) Unprotonated (neutral) charge distribution, illustrating the ligand's polarity and potential interaction zones. (d) Hydrophobic surface, showing nonpolar interactions that contribute to binding stabilization. (e) Acidic and basic surface mapping, identifying proton exchange sites relevant for ionic and hydrogen bonding. (f) A solvent-accessible surface area (SASA) representation with polar interactions highlights the extent of ligand burial within the G-quadruplex structure and reduced solvent exposure, indicating strong and stable binding.

Visual analysis of the docked complex in Discovery Studio (Fig. 16a–f) confirms the multi-modal nature of the interaction.

Fig. 16(a) highlights π–π stacking between the aromatic core and guanine tetrads, a hallmark of quadruplex stabilization.

Fig. 16(b) shows a dense hydrogen-bond network anchoring the drug within the groove and loop regions.

Fig. 16(c) depicts electrostatic potential complementarity—negatively charged DNA phosphate backbone aligning with positively polar drug moieties.

Fig. 16(d) reveals hydrophobic surface interactions reducing solvent exposure.

Fig. 16(e) demonstrates minimal solvent accessibility (low SASA), implying a deeply embedded and stable complex.

Fig. 16(f) illustrates van der Waals surface complementarity, with minimal steric clashes indicating excellent molecular fit.

#### 3.8.5 Mechanistic and biological implications

The combination of strong π–π stacking, hydrogen bonding, electrostatic, and hydrophobic interactions confirms that 1-VAQ stabilizes the G-quadruplex d-[TTAGGGT]_4_ through a multimodal binding mechanism. The docking-derived binding energy and values closely match the experimentally obtained Gibbs free energies (−34 to −37 kJ mol^−1^), validating the computational model. Mechanistically, this compound's stabilization of the G-quadruplex can block telomerase access, reduce telomere elongation and halting oncogenic replication. These *in silico* results support the spectroscopic, electrochemical, and zeta-potential data, collectively showing that the compound is a strong G-quadruplex stabilizer with notable anticancer potential. G-quadruplex stabilizer with significant anticancer potential.

## 4. Conclusion

This study elucidates the binding mechanism between the parallel G-quadruplex DNA sequence d-[TTAGGGT]_4_ and the anthracene-9,10-dione derivative 1-VAQ using an integrated experimental and computational approach. ^1^H NMR spectroscopy provided direct molecular-level evidence of complex formation, showing localized chemical shift perturbations and progressive peak broadening predominantly within the anthraquinone aromatic region. These changes indicate proximity of the aromatic core to the G-quartet surface and support a reversible binding process consistent with external π–π interactions rather than intercalation. Circular dichroism measurements further confirmed that 1-VAQ preserves the characteristic parallel G-quadruplex topology. The enhancement of CD intensity without alteration of the spectral signature indicates structural stabilization of the pre-formed G-quadruplex rather than conformational disruption. UV-Vis spectroscopy revealed pronounced hypochromism accompanied by a slight hypsochromic shift upon ligand addition, consistent with π–π stacking interactions. Fluorescence titration showed concentration-dependent quenching with binding constants on the order of 10^6^ M^−1^, confirming strong and specific association. The absence of significant shifts in the emission wavelength further supports surface binding rather than deep intercalation.

Electrochemical analysis *via* cyclic voltammetry demonstrated redox potential shifts and decreased peak currents upon complexation, reflecting modification of the anthraquinone core's electronic environment and stabilization within the G-quadruplex framework. Zeta-sizer measurements showed an increase in hydrodynamic diameter consistent with complex formation in solution, without evidence of uncontrolled aggregation. Thermodynamic analysis across techniques yielded negative Gibbs free energy values (Δ*G* = −31 to −37 kJ mol^−1^), confirming that spontaneous, energetically favorable binding is dominated by non-covalent interactions. Computational studies align closely with the experimental observations. Molecular docking supports an external end-stacking mode of the anthraquinone plane on terminal G-tetrads, with additional groove-oriented interactions of the sulfonamide side chain. DFT calculations indicate reduced HOMO–LUMO separation and enhanced electronic delocalization upon binding, consistent with the experimentally observed stability. Collectively, these results establish that 1-VAQ stabilizes telomeric G-quadruplex DNA primarily through cooperative π–π stacking and secondary hydrogen-bonding/electrostatic interactions. This stabilization provides a mechanistic basis for potential telomerase inhibition and highlights anthraquinone-based scaffolds as promising templates for rational G-quadruplex-targeted drug design.

## Conflicts of interest

There are no conflicts to declare.

## Data Availability

All data supporting the findings of this study are available within the article.
